# Promoting Healthy Aging Through Policy Change on the Federal Level: Strategies for Scholars and Clinicians

**DOI:** 10.1093/geroni/igaa057.342

**Published:** 2020-12-16

**Authors:** Alison Hernandez, Lieke van Heumen, Luming Li, Marie Gualtieri, Ivorie Stanley, Lisa O’Neill, Nancy Kusmaul

**Affiliations:** 1 Northwestern University, Washington, District of Columbia, United States; 2 University of Illinois at Chicago, Chicago, Illinois, United States; 3 Yale School of Medicine, New Haven, United States; 4 Washington, District of Columbia, United States; 5 University of Arizona, Tucson, Arizona, United States; 6 UMBC, Baltimore, Maryland, United States

## Abstract

Influencing health policy change is a significant focus for scholars and clinicians in the aging field. Nevertheless, they seldom receive formal training on how to influence the policymaking process effectively or how to translate their clinical and research experiences to inform policymakers best. Exposing scholars and clinicians to the policymaking process can advance their effectiveness as they seek to realize meaningful change to promote healthy aging of our population. This poster presentation focuses on providing scholars and clinicians with strategies to understand and influence the federal policymaking process. The presentation addresses the current policy environment in which federal health and aging policy is made and describes challenges to this process. Four strategies are identified to help scholars and clinicians influence the policymaking process: 1) identify a problem and any relevant policy that corresponds to the issue, 2) identify evidence-based solutions that relate to quality improvement, population health, or reducing per capita cost of healthcare, 3) grow relevant networks, meet experts, build relationships and connect with key stakeholders, 4) identify potential unintended consequences or barriers to the implementation of policy. By providing examples, this how-to aging and health policy road map provides context and guidance to stakeholder engagement, frameworks, and methods that can be used to engage in the policymaking process. The final part of this presentation explores ways to integrate health policy training and experience into scholars’ and clinicians’ professional development.

